# The Role of Frontal Assessment Battery and Frontal Lobe Single-Photon Emission Computed Tomography in the Differential Diagnosis of Progressive Supranuclear Palsy Variants and Corticobasal Syndrome—A Pilot Study

**DOI:** 10.3389/fneur.2021.630153

**Published:** 2021-02-04

**Authors:** Piotr Alster, Bartosz Migda, Natalia Madetko, Karolina Duszyńska-Wąs, Agnieszka Drzewińska, Ingeborga Charzyńska, Miłosz Starczyński, Ada Szepelska, Leszek Królicki, Andrzej Friedman

**Affiliations:** ^1^Department of Neurology, Medical University of Warsaw, Warsaw, Poland; ^2^Ultrasound Diagnostic Department, Faculty of Medical Sciences, Medical University of Warsaw, Warsaw, Poland; ^3^Department of Nuclear Medicine and Magnetic Resonance, Mazowiecki Hospital Brodnowski, Warsaw, Poland; ^4^Department of Nuclear Medicine, University Clinical Center, Medical University of Warsaw, Warsaw, Poland

**Keywords:** SPECT, tauopathies, parkinsonism, progressive supranuclear palsy, frontal lobe

## Abstract

Progressive supranuclear palsy (PSP) and corticobasal syndrome (CBS) are clinical syndromes classified as atypical parkinsonism. Due to their overlapping symptomatology, recent research shows the necessity of finding new methods of examination of these clinical entities. PSP is a heterogenic disease. PSP Richardson-Steele Syndrome (PSP-RS) and parkinsonism predominant (PSP-P) are the most common clinical variants of progressive supranuclear palsy syndrome. The different clinical course and life expectancy of PSP-RS and PSP-P stress the need of efficient examination in the early stages. The aim of the study was to evaluate the possible feasibility of the combined use of frontal assessment battery (FAB) and single-photon emission computed tomography (SPECT) in the differentiation of PSP-RS, PSP-P, and CBS. The findings show that FAB may be interpreted as a possible supplementary tool in the differential diagnosis of PSP-P and PSP-RS. The differences in SPECT are less pronounced. The study does not show any advantages of performing combined frontal SPECT and FAB in the differential examination of PSP and CBS. Moreover, PSP-RS and CBS, in a detailed evaluation of the frontal lobe, do not show any significant differences. This is a relatively small study which, however, highlights the relevant features of clinical examination of these rare entities.

## Introduction

The examination of tauopathic atypical parkinsonism remains a difficult issue. The differentiation of progressive supranuclear palsy syndrome and corticobasal syndrome (CBS) is affected by significant overlaps in the diseases' symptomatology. Growing interest is associated with the search for effective tools in the assessment of four-repeat tauopathies and their clinical manifestations (1). The recent criteria of diagnosis of PSP show four critical axes of diagnosis—akinesia, postural instability, cognitive and language deficiencies, and oculomotor dysfunction—and stress the necessity of discriminating variants (2). Among the variants of PSP, the most common—PSP-Richardson–Steele syndrome and PSP-parkinsonism predominant—should be primarily indicated as they are related with up to 90% cases of PSP (about 60% of PSP-RS and about 30% of PSP-P)^3^. Additionally, recent literature highlights the boundaries between PSP and CBS and stresses the need for finding examination tools, which may be supplemental to neurological examination and most common additional assessments such as magnetic resonance imaging (MRI) (1, 3–5). The contemporary criteria of diagnosis of CBS were released in 2013 and do not explore the field of evolving supplementary examinations (3). The studies based on positron emission tomography (PET) showed various limitations as off-binding of radiotracer observed in [^18^F]-AV1451-PET, non-specific radiotracers as [^18^F]-FDG-PET^7^, or unfortunate economical aspect. The second-generation tau radiotracers such as [^18^F]-PI2620-PET seem to play a possibly beneficial role; however, they are not accessible in everyday clinical practice. Single-photon emission computed tomography (SPECT), with its various radiotracers such as ^99m^Tc-HMPAO, is more accessible which, however, is affected by low specificity. Previous studies with SPECT-^99m^Tc-HMPAO conducted on patients with tauopathic atypical parkinsonism showed thalamic hypoperfusion in PSP which, however, did not confirm any significant differences of perfusion between PSP and CBS (6, 7). A combined assessment using dopamine transporter and perfusion SPECT was evaluated in a paper by Van Laere et al. where the authors attempted to define the role of this assessment in the differential diagnosis of parkinsonism (8). The study examined patients with diagnosis of idiopathic Parkinson's disease, essential tremor, PSP, multiple-system atrophy, and dementia with Lewy bodies. The singular dopamine transporter evaluation enabled 58.8% effective differentiation. Perfusion examination presented effectiveness in 67.6% of differential diagnoses. The combined examination showed 82.4% efficacy. The study was based on the examination of small groups−12 PSP patients (8). The authors did not discuss PSP phenotypes as separate entities. The issue of combined perfusion, metabolism, and dopaminergic evaluation was earlier evaluated in PET. Striatal abnormalities in metabolic PET were found to be sensitive in the examination of multiple system atrophy (MSA); however, dopaminergic evaluation was not found to be feasible in the differential examination of parkinsonian syndrome (9). Another work presented the differentiation of parkinsonism using technetium-99m ethyl cysteinate dimer. It confirmed a potentially beneficial role in the differential diagnosis of MSA and idiopathic Parkinson's disease (PD) (10). An examination performed using simultaneous ^99m^Tc-ECD/^123^I-FP-CIT revealed higher striatal binding in MSA when compared to PD. Asymmetry was more prominent in PD^13^. A study evaluating Tc-99m ethylene cysteinate in the SPECT examination of PD and MSA showed elevated perfusion in the lentiform, cerebellum, and thalamus among patients with PD (11).

The diagnosis of PSP-P was not stressed in any of the studies. Regarding the limited feasibility of SPECT in the examination of tauopathic parkinsonism, the authors of this study intended to verify the usefulness of combined examination using assessment of frontal lobe in perfusion and neuropsychological assessment using frontal assessment battery (FAB), a short screening test that evaluates the executive functions.

According to the most recent theories, frontal lobes are responsible for the control of complex functions, such as abstract reasoning, self-regulation, motor programming, mental flexibility, inhibitory control, and environmental autonomy (12). Assessing these functions and being able to identify the dysexecutive syndrome are helpful for the diagnosis of brain diseases, such as frontotemporal dementia, parkinsonian dementia, and vascular dementia.

Deficits in executive functioning may be observed in PD and also in all atypical parkinsonisms (13–15). The severity of the dysexecutive syndrome in these diseases may vary from mild deterioration to a highly pronounced executive dysfunction being one of the main symptoms (PSP-RS). It may also coexist with other cognitive deficits (as in some manifestations of CBS) (15). However, as present in almost all patients with parkinsonism, the dysexecutive syndrome should be always neuropsychologically assessed. Several neuropsychological tests and clinical trials were designed to assess the frontal lobe functions. The most known and widely used are The Wisconsin Card Sorting Test (WCST), the Stroop Test, the Tower of London, Brixton Spatial Anticipation Test, and the Behavioral Assessment of the Disexecutive Syndrome (BADS) (13). All of them are tests of confirmed sensitivity to the disexecutive syndrome; however, they assess just some several aspects of executive functioning (WCST and Stroop), take quite a lot of time, and require some more complex preparations and use of test tools (BADS) or the results depend on the time of performance (ToL).

FAB is designed to be administered at bedside in about 10 min. It consists of six tasks, each of which was designed to assess one of the main frontal lobe functions (abstract reasoning, mental flexibility, motor programming, inhibitory control, sensitivity to interference, environmental autonomy)^15^. It is possible to receive zero to three points for each of the test items, giving a maximal total score of 18 points. None of the tasks requires any tools. There is no need for the patient to be able to perform complex movements (which is particularly important while assessing patients with movement disorders, such as Parkinson's Disease or atypical parkinsonian syndromes) (13). The FAB has been found to highly correlate with the results of other neuropsychological tests measuring executive functions (e.g., WCST) and is known for its sensitivity to executive dysfunctions in parkinsonism (14), which makes it a useful tool for clinical practice.

## Materials and Methods

In this prospective study, all patients gave informed consent to participate in this research. The bioethical committee of the Medical University of Warsaw approved this study. From May 2017 to September 2020, 58 patients, in total, were enrolled. The neurological examination and diagnosis were based on the recent criteria and conducted in the Department of Neurology of the Medical University of Warsaw in all of the cases. The neuropsychological examination was performed by two neuropsychologists working (9 years of experience) in the Department of Neurology at the Medical University of Warsaw and experienced in the assessment of psychological deficiencies in atypical parkinsonism.

Due to the fact that certain patients did not accomplish the examination for various reasons, the authors of this study were forced to exclude about 29.3% of the cases primarily planned for further evaluation. Finally, the research group was based on 41 participants with clinical diagnosis of probable PSP-P, CBS, and PSP-RS and consisted of 18 patients with PSP-RS (11 male, seven female), 11 patients with PSP (six male, five female), and 12 patients with CBS (one male, 11 female). All patients were right-handed, and the duration of the disease varied from 2 to 5 years. Out of the 41 study participants, 23 (56.1%) were female and 18 (43.9%) were male. The mean age was 70.2 years (range, 54–85 years) (Table 1).

**Table 1:** Basic characteristics of research group and subgroups: progressive supranuclear palsy-Richardson–Steele syndrome (PSP-RS), progressive supranuclear palsy-parkinsonism predominant (PSP-P), and corticobasal syndrome (CBS) in relation to single-photon emission computed tomography parameters, frontal assessment battery, and subgroups comparison.

	**All** ***N*** **=** **41**					**PSP–RS** ***N*** **=** **18**					**PSP–P** ***N*** **=** **11**					**CBS** ***N*** **=** **12**					***p*** **PSP–RS vs**. **PSP–P**	***p*** **PSP–RS vs**. **CBS**	***p*** **PSP–P vs**. **CBS**
	**F/M**	**Mean**	**Min**	**Max**	**SD (95% CI)**	**F/M**	**Mean**	**Min**	**Max**	**SD (95% CI)**	**F/M**	**Mean**	**Min**	**Max**	**SD (95% CI)**	**F/M**	**Mean**	**Min**	**Max**	**SD (95% CI)**			
Gender	23/18					7/11					6/5					11/1					0.7276c	0.0121cy	0.0509cy
Age		70.2	54.0	85.0	6.8 (5.6–8.7)		71.1	59	80	5.7 (4.3–8.5)		70.2	57	77	6.9 (4.8–12.1)		68.9	54	85	8.5 (6–14.5)	0.713t	0.4151t	0.7009t
FAB		12.3	6.0	18.0	2.9 (2.4–3.7)		11.4	6	18	2.9 (2.2–4.3)		13.9	11	17	1.9 (1.3–3.4)		12.3	7	16	3.2 (2.2–5.4)	0.0165t	0.4481t	0.1481t
(1) Frontal lobe		−1.7	−6.9	2.6	1.8 (1.5–2.4)		−1.8	−6.9	1.5	1.9 (1.4–2.8)		−0.7	−3.5	2.6	2.1 (1.5–3.7)		−2.4	−3.9	0.4	1.2 (0.9–2.1)	0.1765t	0.2718t	0.0231t
(2) Frontal lobe (AAL)		−1.6	−7.2	2.5	1.8 (1.5–2.3)		−1.8	−7.2	1.4	1.9 (1.4–2.8)		−0.6	−3.4	2.5	2 (1.4–3.5)		−2.3	−3.7	0.7	1.2 (0.8–2)	0.107t	0.433t	0.0202t
(16) Frontal lobe (AAL) (L)		−1.5	−6.1	2.6	1.8 (1.5–2.3)		−1.7	−6.1	2	1.8 (1.4–2.8)		−0.4	−3	2.6	1.9 (1.4–3.4)		−2.3	−3.8	0.9	1.2 (0.9–2.1)	0.1056u	0.1624u	0.0138u
(3) Frontal lobe (AAL) (R)		−1.7	−8.1	3.1	2 (1.6–2.5)		−1.9	−8.1	1.6	2.1 (1.5–3.1)		−0.8	−3.6	3.1	2.2 (1.5–3.8)		−2.2	−5.1	0.4	1.5 (1.1–2.6)	0.3012u	0.5117u	0.1962u
(4) Frontal lobe-Atlas 2		−2.8	−9.6	2.3	2.3 (1.9–3)		−2.7	−9.6	1.6	2.5 (1.8–3.7)		−1.9	−5.2	2.3	2.5 (1.7–4.3)		−3.6	−6.1	0.7	1.8 (1.2–3)	0.417t	0.252t	0.0634t
(5) Frontal lobe-Atlas 1		−1.3	−9.4	2.8	2.3 (1.9–2.9)		−1.2	−9.4	1.8	2.6 (2–3.9)		−0.4	−4.5	2.8	2.3 (1.6–4)		−2.1	−4	1	1.6 (1.1–2.7)	0.4448u	0.049u	0.0694u
(17) Frontal lobe-Atlas 3		−2.4	−9.3	2.2	2.2 (1.8–2.8)		−2.5	−9.3	1.2	2.4 (1.8–3.5)		−1.4	−5.1	2.2	2.4 (1.7–4.3)		−3.3	−5.2	0.1	1.4 (1–2.3)	0.236t	0.3317t	0.0338t
(6) Inferior frontal gyrus, opercular part (AAL) (L)		−2.6	−6.0	5.2	2.2 (1.8–2.9)		−2.4	−5.8	0.5	1.9 (1.4–2.8)		−1.9	−4.3	1.7	1.9 (1.3–3.3)		−3.4	−6	5.2	2.9 (2.1–5)	0.6531u	0.0515u	0.0228u
(7) Inferior frontal gyrus, opercular part (AAL) (R)		−1.5	−5.6	4.8	2.2 (1.8–2.8)		−1.5	−5.1	2.1	2 (1.5–3)		−1.2	−5	4.8	2.6 (1.8–4.6)		−1.7	−5.6	3.1	2.2 (1.5–3.7)	0.7487t	0.8227t	0.6556t
(8) Inferior frontal gyrus, orbital part (AAL) (L)		−2.1	−6.4	3.1	2.4 (2–3.1)		−2.4	−6	1.7	2.2 (1.6–3.3)		−1.7	−5.7	1	1.9 (1.3–3.4)		−2	−6.4	3.1	3.1 (2.2–5.3)	0.3903t	0.7089t	0.764t
(9) Inferior frontal gyrus, orbital part (AAL) (R)		−1,0	−5.4	4.6	2.1 (1.7–2.7)		−1.2	−5.4	3.2	2.1 (1.6–3.2)		−1.1	−5.1	1.6	2 (1.4–3.4)		−0.7	−4.2	4.6	2.2 (1.6–3.8)	0.8667t	0.5015t	0.6415t
(10) Inferior frontal gyrus, triangular part (AAL) (L)		−1.4	−5.5	2.5	2.3 (1.9–2.9)		−1.1	−5.5	2.5	2.6 (1.9–3.8)		−1.5	−3.9	1.7	1.9 (1.3–3.4)		−1.8	−5.3	2.1	2.3 (1.7–4)	0.7112t	0.4566t	0.6913t
(18) Inferior frontal gyrus, triangular part (AAL) (R)		−0.5	−5.3	4.4	2.3 (1.9–2.9)		−0.3	−2.9	4.2	1.9 (1.5–2.9)		−0.9	−5.3	3.4	2.9 (2–5.1)		−0.5	−4.7	4.4	2.3 (1.6–3.9)	0.5684t	0.8298t	0.7528t
(11) Middle frontal gyrus (AAL) (L)		−1,0	−3.7	3.6	1.7 (1.4–2.1)		−0.8	−3.6	3.6	1.9 (1.4–2.8)		−0.4	−2.7	2.6	1.5 (1.1–2.7)		−1.9	−3.7	0.4	1.1 (0.8–1.9)	0.5015t	0.075t	0.0106t
(12) Middle frontal gyrus (AAL) (R)		−1.7	−8.5	3.9	2.4 (1.9–3)		−1.5	−8.5	1.2	2.2 (1.7–3.4)		−0.9	−3.3	3.9	2.4 (1.7–4.3)		−2.7	−6.5	0.4	2.3 (1.6–3.9)	0.9105u	0.1384u	0.1569u
(13) Middle frontal gyrus, orbital part (AAL) (L)		−1.3	−5.7	3.4	2.3 (1.9–2.9)		−0.8	−5.7	3.4	2.6 (2–3.9)		−1.2	−4.1	1.8	1.9 (1.3–3.3)		−2.3	−5.3	1.9	1.9 (1.4–3.3)	0.6958t	0.111t	0.1822t
(14) Middle frontal gyrus, orbital part (AAL) (R)		−1.8	−5.2	3.1	2.1 (1.8–2.7)		−1.9	−5.2	3.1	2.4 (1.8–3.6)		−1.3	−3.9	2.5	1.8 (1.3–3.2)		−2	−4.1	3.1	2.1 (1.5–3.5)	0.486u	0.8989u	0.1481u
(15) Superior frontal gyrus, dorsolateral (AAL) (L)		−1.1	−5.7	3.3	2 (1.7–2.6)		−1.1	−5.1	2.9	2 (1.5–3)		0.3	−3.4	3.3	2 (1.4–3.5)		−2.2	−5.7	−0.3	1.4 (1–2.4)	0.0803t	0.1344t	0.0027t
(5) Superior frontal gyrus, dorsolateral (AAL) (R)		−1.2	−6.2	3.1	1.8 (1.5–2.4)		−1.4	−6.2	0.9	1.8 (1.3–2.7)		−0.2	−3.9	3.1	2 (1.4–3.5)		−1.9	−4.4	0.1	1.4 (1–2.4)	0.0953t	0.4544t	0.0268t
(19) Superior frontal gyrus, medial (AAL) (L)		−1.1	−3.9	1.5	1.3 (1–1.6)		−1.5	−3.9	0.5	1.2 (0.9–1.8)		−0.2	−1.8	1.5	1.1 (0.8–1.9)		−1.4	−2.9	1.1	1.2 (0.8–2)	0.0091t	0.9354t	0.0171t
(20) Superior frontal gyrus, medial (AAL) (R)		−1.2	−6.9	2.2	1.7 (1.4–2.2)		−1.6	−6.9	0.4	1.7 (1.3–2.6)		−0.3	−3.7	2.2	1.9 (1.3–3.3)		−1.3	−2.9	1.7	1.4 (1–2.3)	0.1009u	0.9325u	0.1397u
(21) Superior frontal gyrus, medial orbital (AAL) (L)		−1.2	−10.4	2.6	2.5 (2–3.1)		−1	−10.4	2.2	2.9 (2.2–4.3)		−0.6	−4.3	2.6	1.9 (1.3–3.3)		−2.1	−5.1	2.6	2.1 (1.5–3.6)	0.8047u	0.072u	0.0489u
(22) Superior frontal gyrus, medial orbital (AAL) (R)		−0.7	−8.3	3.4	2.2 (1.8–2.9)		−0.6	−8.3	2	2.4 (1.8–3.6)		−0.3	−3.5	3.4	2.1 (1.4–3.6)		−1.3	−4	2.7	2.2 (1.6–3.7)	0.9105u	0.0904u	0.2184u
(23) Superior frontal gyrus, orbital part (AAL) (L)		−0.7	−8.1	3.1	2 (1.7–2.6)		−0.7	−8.1	2.9	2.3 (1.8–3.5)		−0.4	−4.5	2.3	1.7 (1.2–3)		−1.1	−3.2	3.1	2 (1.4–3.3)	0.7192u	0.2276u	0.1569u
(24) Superior frontal gyrus, orbital part (AAL) (R)		−1.5	−11.6	2.6	2.3 (1.9–3)		−1.6	−11.6	2.2	3.1 (2.3–4.6)		−1.2	−4	1	1.4 (1–2.4)		−1.4	−3.5	2.6	1.9 (1.3–3.2)	0.9642u	0.6567u	0.5588u

*SD, standard deviation; CI, confidence interval*.

*For p-value: “t” for Student's t test, “u” for Mann–Whitney U test, “c” for χ^2^ test, “cy” for χ^2^ square test with Yates correction*.

The final research group underwent neuropsychological examination with FAB testing and perfusion assessment using SPECT ^99m^Tc-HMPAO. Due to the fact that the software used in the study to assess perfusion in SPECT shows the results of patients compared to 20 healthy volunteers, due to ethical reasons, SPECT was not additionally conducted on the controls in this study. In order to avoid examining the controls only in neuropsychological examination, the results of the FAB test were compared with the standard results of healthy volunteers from the literature.

### Frontal Assessment Battery

In this study, FAB was used due to the relevant role of frontal lobe syndrome in the symptomatology of PSP. The frontal lobe syndrome is generally associated with the Richardson–Steele variant of PSP, as patients affected by this disease often present rapidly progressing changes in behavior. In this context, FAB, regarding its simplicity and possible screening value, may be interpreted as a valuable supplement in the examination of PSP. As growing interest is related to boundaries between parkinsonian syndromes based on four-repeat tauopathies, in the opinion of the authors of the study, extended evaluation of similarities and differences regarding the frontal lobe in PSP-RS, PSP-P, and CBS seem to be an intriguing issue.

### Single-Photon Emission Computed Tomography

SPECT, with technetium-99m hexamethylpropyleneamine oxime (^99m^Tc-HMPAO) as a radiotracer, was used for the evaluation of regional cerebral blood flow. Then, 740 MBq of radiotracer was administered in patients placed in a quiet, dimly lit room in supine position. Examinations were performed with SPECT/CT scan (Symbia T6, Siemens) on dual-head gamma camera with low-energy high-resolution parallel-hole collimator. Step and shoot acquisition mode was used, and sequences of 128 frames on a 128 × 128 matrix were obtained (64 projections per head, 30 s per projection). The photopeak was set at 140 keV with 10% window on either site of the photopeak. Iterative reconstruction (eight iterations, eight subsets, 7 mm Gauss filter), scatter correction, and CT attenuation correction were performed. Post-processing analysis was performed with Scenium software (Siemens Medical Solutions USA, Inc.). The regions of interest (ROIs) were predefined on a high-resolution T1 MRI volume scan. Perfusion in the basal ganglia, frontal lobes, hemispheres of cerebella, and thalami was subsequently examined among all patients. Values of variances from ROIs in individual parts of the frontal lobe on both sides (right and left separately) were taken for statistical analysis.

### Statistical Analysis

Statistical analyses were performed using Statistica software (version 13.1, Dell, Inc. Statsoft). The presented data were expressed as means with 95% confidence interval. Data distributions were assessed by Shapiro–Wilk *W* test. For comparison of parametric and non-parametric variables, Student's *t* test and Mann–Whitney *U* test were used, respectively. Frequencies of nominal variables were compared using χ^2^ test. In case of small group counts, Yates correction was used. We performed receiver operating characteristic (ROC) curves to evaluate the diagnostic performance of SPECT parameters and FAB as predictors of PSP-RS, PSP-P, and CBS analyzing sensitivity and specificity for each possible threshold/cutoff, and we used area under the ROC curve (AUC) to express the overall diagnostic accuracy of the index criterion and for comparison between significant parameters. Analysis was made in search of the parameters that best differentiate particular subgroups against each other. We have reported 95% confidence interval for calculated AUC *p*-value. Based on the ROC curves, we have determined the cutoff point for each parameter and reported its positive predictive value (PPV), negative predictive value (NPV), and accuracy (ACC). Those results were used in next-step multivariable analysis. For this purpose, we have used logistic regression to answer a question if any combination of SPECT parameters and FAB has greater overall performance in relation to single-variable analysis in differentiating PSP-RS, PSP-P, and CBS with a report of OR and its 95% confidence interval, accuracy, and level of significance (*p*-value). *P* < 0.05 was considered as indicative of a statistically significant difference. In the logistic regression part of the analysis, we have made an effort to build a multivariate model characterizing each of the subgroups separately using logistic regression and taking into account previous results.

## Results

### Basic Characteristics

The mean, maximal, minimal, and standard deviation with 95% confidence interval values of age, frontal assessment battery, and SPECT parameters [divided into right (R) and left (L) sides] are listed in Table 1. A comparison of PSP-P and PSP-RS revealed significantly higher values of FAB for PSP-P (13.9 vs. 11.4; *p* = 0.0165) and higher values of SPECT variances [superior frontal gyrus, medial (AAL) on the left side] for PSP-P (−0.2 vs. −1.5; *p* = 0.0091; Table 1). Higher values of SPECT variances were also obtained for PSP-P in relation to CBS in several regions: frontal lobe, −0.7 vs. −2.4 (*p* = 0.023); frontal lobe (AAL), −0.6 vs. −2.3 (*p* = 0.0202); frontal lobe (AAL) (L), −0.4 vs. −2.3 (*p* = 0.0138); frontal lobe (flutemetamol), −1.4 vs. −3.3 (*p* = 0.0338); inferior frontal gyrus opercular part (AAL) (L), −1.9 vs. −3.4 (*p* = 0.0228); middle frontal gyrus (AAL) (L), −0.4 vs. −1.9 (*p* = 0.0106); superior frontal gyrus, dorsolateral (AAL) (L), 0.3 vs. −2.2 (*p* = 0.0027); superior frontal gyrus, dorsolateral (AAL) (R), −0.2 vs. −1.9 (*p* = 0.0268); superior frontal gyrus, medial (AAL) (L), −0.2 vs. −1.4 (*p* = 0.0171); and superior frontal gyrus, medial orbital (AAL) (L), −0.6 vs. −2.1 (*p* = 0.0489) (Table 1). Assessment of SPECT parameters in relation to PSP-RS and CBS revealed significant differences only in one region, frontal lobe—Atlas 1, with higher values of SPECT variances in the case of PSP-RS, −1.2 vs. −2.1 (*p* = 0.049) (Table 1).

### ROC Curve Analysis

**Figure 1:**
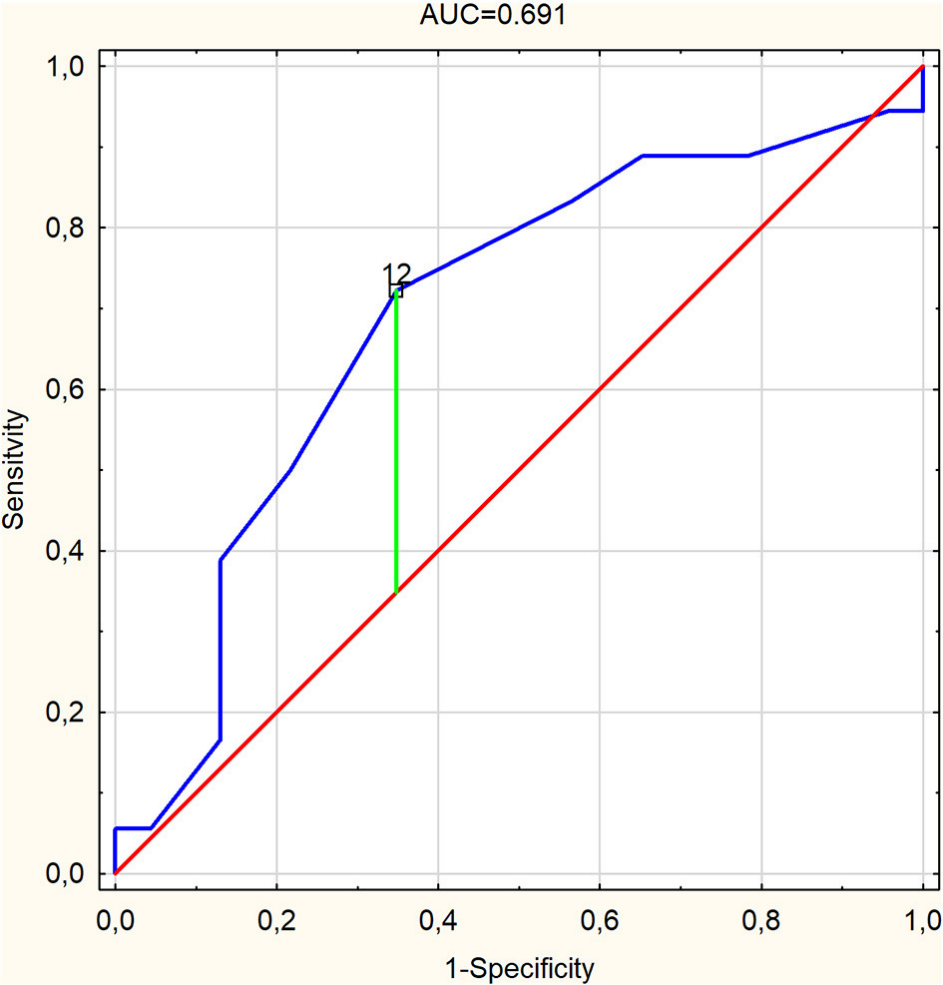
Receiver operating characteristic curve graph of frontal assessment battery (area under the ROC curve = 0.691) as a predictor for progressive supranuclear palsy-Richardson–Steele syndrome with marked cutoff value.

**Figure 2:**
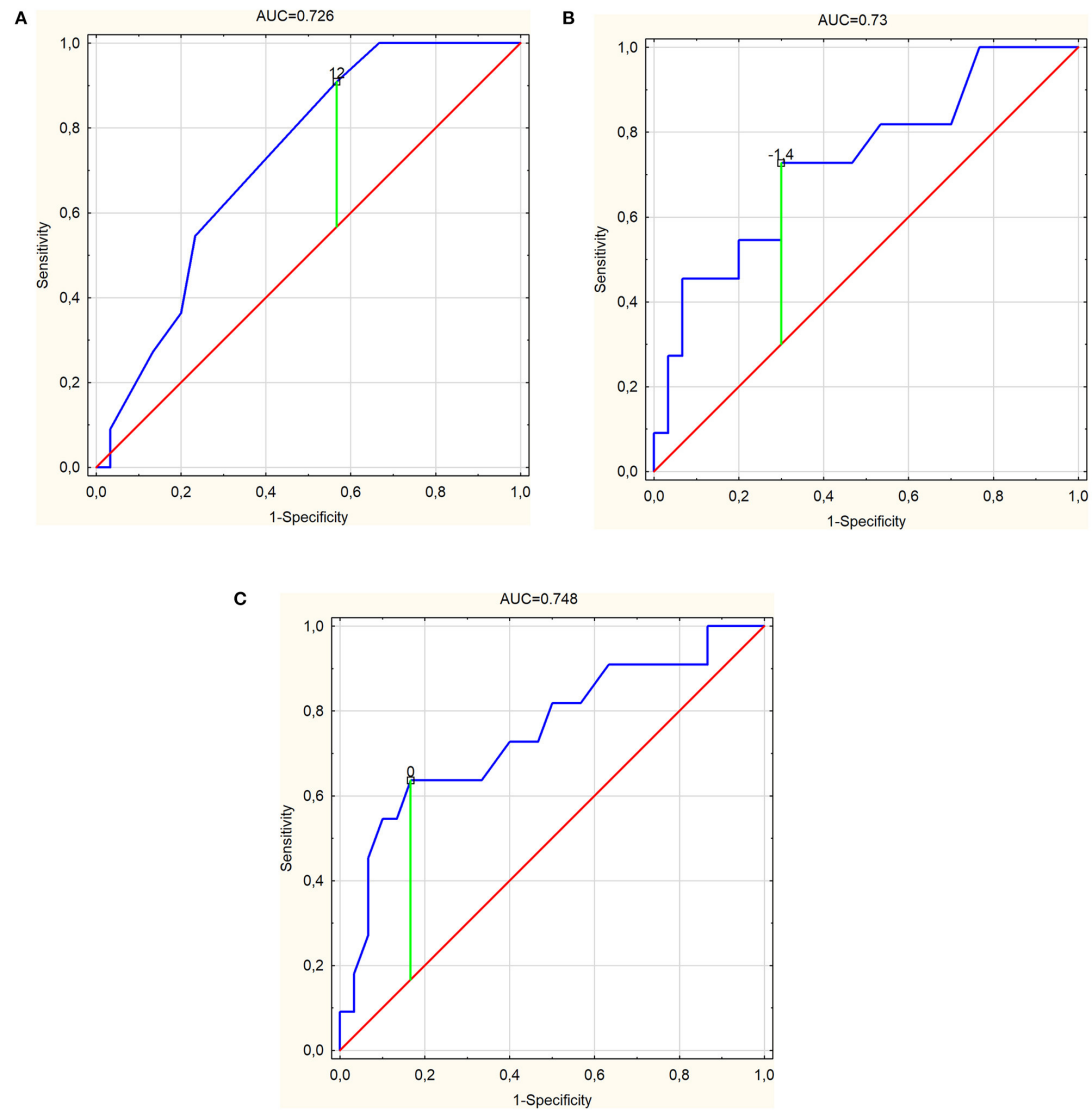
Receiver operating characteristic curve graphs of **(A)** frontal assessment battery [area under the ROC curve (AUC) = 0.726], **(B)** frontal lobe (AAL) (L) (AUC = 0.73), and **(C)** superior frontal gyrus, dorsolateral (AAL) (L) (AUC = 0.748) as independent predictors for progressive supranuclear palsy-parkinsonism predominant with marked cutoff values.

**Figure 3:**
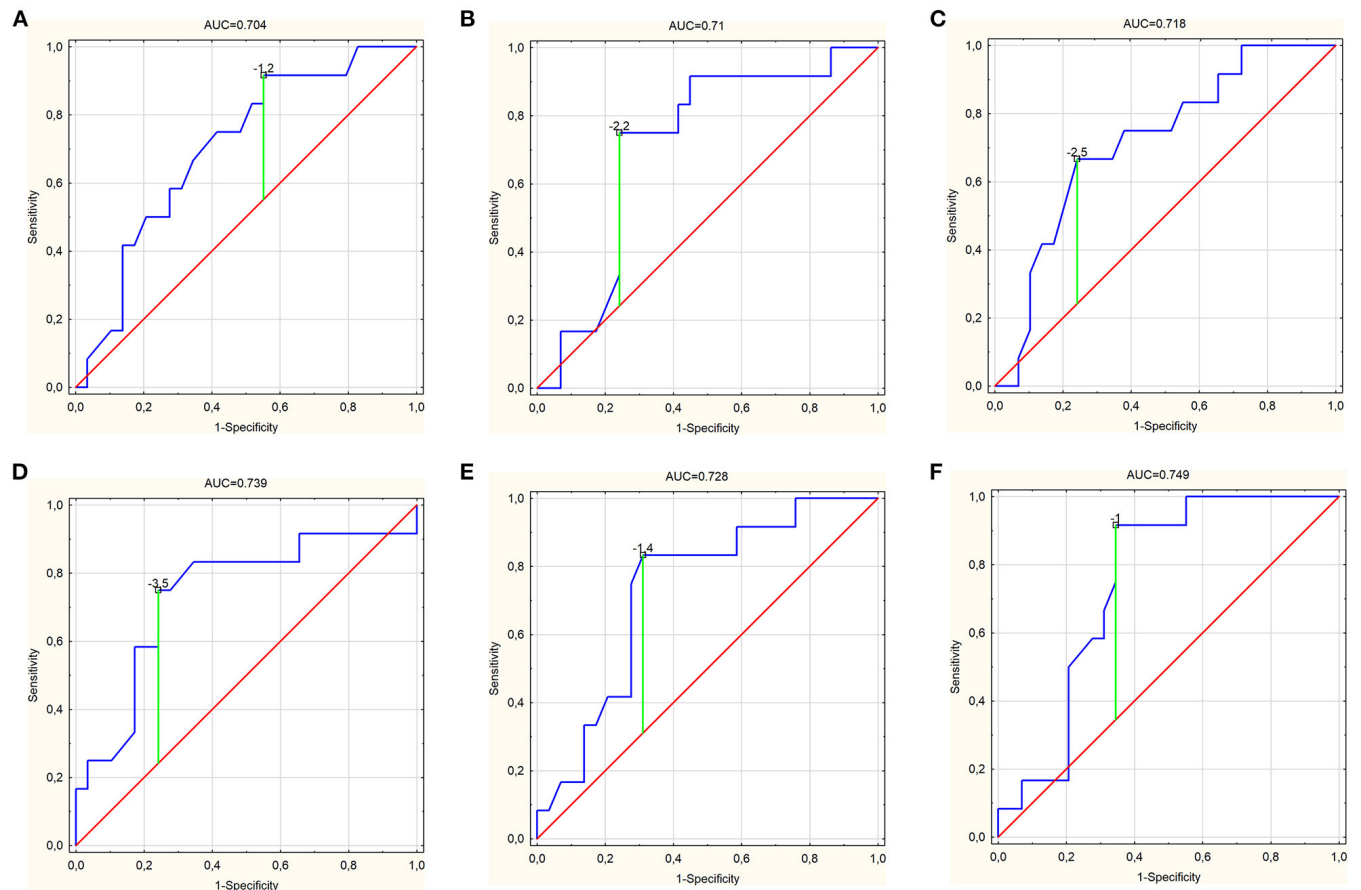
Receiver operating characteristic curve graphs of **(A)** frontal lobe [area under the ROC curve (AUC) = 0.704], **(B)** frontal lobe (AAL) (L) (AUC = 0.71), **(C)** frontal lobe—Atlas 1 (AUC = 0.718), **(D)** inferior frontal gyrus, opercular part (AAL) (L) (AUC = 0.739), **(E)** middle frontal gyrus (AAL) (L) (AUC = 0.728), and **(F)** superior frontal gyrus, dorsolateral (AAL) (L) (AUC = 0.749) as independent predictors for corticobasal syndrome with marked cutoff values.

In the case of PSP-RS, only the FAB turned out to be a significant parameter differentiating this subgroup from the others with AUC of 0.691 (95% CI, 0.522–0.86; *p* = 0.027) and cutoff of 12, with sensitivity, specificity, PPV, NPV, and ACC at 72.2, 65.2, 61.9, 75, and 68.3%, respectively (Table 2 and Figure 1). Similarly, for PSP-P, among others, FAB turned out to be a significant parameter, with AUC of 0.726 (95% CI, 0.568–0.883; *p* = 0.0049) and the same cutoff of 12 and with higher values of sensitivity at 90.9% and NPV at 92.9% but lower specificity, PPV, and ACC at 43.3, 37, and 56.1%, respectively (Figure 2A). The other parameters include frontal lobe (AAL) L (Figure 2B) and superior frontal gyrus dorsolateral (AAL) L (Figure 2C), with higher values of AUC at 0.73 (95% CI, 0.552–0.909; *p* = 0.0115) and 0.748 (95% CI, 0.567–0.93; *p* = 0.0073), respectively, and a slightly better overall performance (Table 2). For CBS, the essential parameters occurred to be six SPECT parameters as listed in Table 1 and Figures 3A–F. The best overall performance revealed frontal lobe (AAL) L with AUC = 0.71 (95% CI, 0.542–0.878; *p* = 0.0145) and with cutoff value equal to −2.2 and sensitivity, specificity, PPV, NPV, and ACC at 75, 95.7, 90, 88, and 88.6%, respectively (Figure 3B). The highest value of AUC (0.749; 95% CI, 0.601–0.896; *p* = 0.001) was calculated for superior frontal gyrus dorsolateral (AAL) L (Figure 3F), with slightly higher values of sensitivity and NPV but with lower values of specificity, PPV, and ACC (Table 2).

**Table 2:** Receiver operating characteristic curve analysis of single-photon emission computed tomography parameters and frontal assessment battery (FAB).

		**S/D**	**Cutoff**	**AUC**	**95% CI**	***p***	**Se**	**Sp**	**PPV**	**NPV**	**ACC**
FAB	PSP-RS	D	12	0.691	0.522–0.86	0.027	72.2	65.2	61.9	75	68.3
FAB	PSP-P	S	12	0.726	0.568–0.883	0.0049	90.9	43.3	37	92.9	56.1
(16) Frontal lobe (AAL) (L)		S	−1.4	0.73	0.552–0.909	0.0115	72.7	70	47.1	87.5	70.7
(15) Superior frontal gyrus, dorsolateral (AAL) (L)		S	0	0.748	0.567–0.93	0.0073	63.6	83.3	58.3	86.2	78
(1) Frontal lobe	CBS	D	−1.2	0.704	0.536–0.872	0.0171	91.7	44.8	40.7	92.9	58.5
(16) Frontal lobe (AAL) (L)		D	−2.2	0.71	0.542–0.878	0.0145	75	95.7	90	88	88.6
(5) Frontal lobe—Atlas 1		D	−2.5	0.718	0.552–0.885	0.0103	66.7	75.9	53.3	84.6	73.2
(6) Inferior frontal gyrus, opercular part (AAL) (L)		D	−3.5	0.739	0.557–0.92	0.0102	75	75.9	56.3	88	75.6
(11) Middle frontal gyrus (AAL) (L)		D	−1.4	0.728	0.566–0.891	0.0058	83.3	69	52.6	90.9	73.2
(15) Superior frontal gyrus, dorsolateral (AAL) (L)		D	−1	0.749	0.601–0.896	0.001	91.7	65.5	52.4	95	73.2

*S/D, stimulant/destimulant; AUC, area under the ROC curve; CI, confidence interval; p, p value for AUC; Se, sensitivity; Sp, specificity; PPV, positive predictive value; NPV, negative predictive value; ACC, accuracy*.

### Logistic Regression

Unfortunately, for PSP-RS, it was not possible to build a model based on logistic regression in any combination of the available variables. In the case of PSP-P and CBS, models were successfully built, but only based on single variables. Any other combination and adding of the next variables did not bring any statistically significant changes. For PSP-P, the FAB turned out to be an important parameter, with OR of 29.3 (95% CI, 2.6–336.4; *p* = 0.0311) and with diagnostic accuracy of 72.4%. On the other hand, superior frontal gyrus dorsolateral (AAL) L was an important parameter for CBS with OR of 6.0 (95% CI, 1.1–33.4; *p* = 0.0218) and with diagnostic accuracy of 82.6% (Table 3).

**Table 3:** Logistic regression analysis of progressive supranuclear palsy-parkinsonism predominant (PSP-P) and corticobasal syndrome (CBS) in relation to single-photon emission computed tomography and frontal assessment battery parameters.

		**OR**	**95% CI**	***p***	**ACC**
FAB	PSP-P	29.3	2.6–336.4	0.0311	72.4
(15) Superior frontal gyrus, dorsolateral (AAL) (L)	CBS	6.0	1.1–33.4	0.0218	82.6

*p for logistic regression*.

*OR, odds ratio; CI, confidence interval; ACC, accuracy*.

## Discussion

### PSP-P as an Important Entity in Differential Diagnosis

To the best of our knowledge, this is the first study to evaluate the examination of frontal lobe as a possible factor differentiating variants of PSP in a combined neuropsychological and perfusion assessment perspective. Our data confirm the clinical variability among patients with the two main subtypes of PSP. As the obtained results show, the impairment in executive functions could be a significant factor in the differential diagnosis of PSP variants. The analysis of the results indicate that the dysexecutive syndrome in the parkinsonian variant (PSP-P) might be less severe than in PSP-RS, with the FAB scores oscillating rather above 12 in the first and under 12 in the latter. Such differences could be correlated to distinct tau distribution in the course of PSP in each of its variants (5). Our findings are congruent with observations made by Pellicano et al. who reported some significant differences in executive functioning in PSP-P vs. PSP-RS patients (19). The lack of additional role of combined examination in FAB and SPECT seems to be a consequence of the limited specificity of the methods and the assessment being limited to the frontal lobe rather than the lack of differences between these two entities. Previous studies showed more severe volume loss in PSP-RS within the frontal pole and inferior frontal gyrus in the volumetric analysis (5). In our study, significant differences between PSP-P and PSP-RS were observed within the superior frontal gyrus medial of the dominant hemisphere. It should also be stressed that, in the majority of works, the abnormalities in PSP-P were rarely observed within the frontal lobe (16). Additionally, more differences were observed in the comparison of perfusion of PSP-P and CBS. This observation, in association with the lack of significant differences between PSP-RS and CBS, shows that perfusion in PSP-P is least deteriorated when evaluating all three entities. The results comparing PSP-P and PSP-RS, on one hand, come up with the findings showing a more beneficial course of the disease and the necessity of evaluating these variants as separate entities (20). The obtained results show that more research in the field involving larger groups of patients should be conducted. Previous studies of SPECT in PSP-P and PSP-RS did not highlight the issue of perfusion; however, an analysis concerning the dopaminergic degeneration in both entities was conducted. In a study evaluating groups of patients with PSP-P—four patients, PSP-RS—six patients, and PD−10 patients, the authors used alternative sets of SPECT—dopamine transporter and I-iodobenzamide D2 receptor radiotracer in each case. The first radiotracer showed significant differences in the putamen-to-caudate ratio between PD and PSP groups (without discriminating variants of PSP). The radiotracer indicating D2 receptor was found to be feasible in the differentiation of PSP-RS and PSP-P as the striatal uptake was reduced in PSP-RS and mildly increased in PSP-P (21). The obtained results, though based on relatively small groups of patients, show that assessment in executive functions using FAB is possibly useful in the differential diagnosis of PSP-P and PSP-RS with similar durations.

### The Boundaries Between PSP-RS and CBS

This study, though presenting minor differences between PSP-RS and CBS, shows that FAB and assessments of perfusion in SPECT present slightly more severe deterioration in CBS. The differences cannot be interpreted as evident as the significant differences in SPECT were observed only in one of the parameters, frontal lobe—Atlas 1 (an area automatically indicated by Scenium software). All other evaluations of the frontal lobe in SPECT did not provide significant differences. This difference does not significantly impact the clinical manifestation and the doubtful boundaries between PSP-RS and CBS. This could be partially explained by similarities of perfusion in the vast majority of ROIs. The combined assessment using FAB and SPECT examination of frontal perfusion does not provide an additional tool in differential diagnosis. The finding confirms the questionable boundaries between the clinical syndromes (1, 7).

However, considering the variety of possible manifestations of CBS, a further research considering the use of other neuropsychological assessment methods should be done. The frontal–executive variant may, in fact, be hardly distinguished from PSP-RS. The other variants of CBS though (the variants with apraxia, aphasia, or visuospatial deficits dominating the clinical manifestation) (13, 15) should be distinguished.

Earlier evaluations of PSP and CBS generally did not discriminate the variant PSP-RS, which could deviate possible findings. In a study analyzing iodine-123-labeled FP-CIT SPECT, the authors indicated high sensitivity in the examination of PSP (22). The work did not take into account the heterogeneity of PSP. A work examining patients with PD, MSA-P, and PSP showed no significant differences between PD and PSP (23). The utility of dopamine transporter SCAN (DaTSCAN) is interpreted as limited in the examination of PSP. The limitations of DaTSCAN in the examination of PSP are related with the lack of differential impact in the examination of Parkinson's disease and atypical parkinsonism (24). The DaTSCAN of a patient with PSP and CBS was also found to lack differences with that of a patient affected by progressive apraxia of speech (25).

### Limitations

This study is based on the examination of relatively small groups of patients, which is a result of examining rare entities in a single department. The authors of this study are aware that the methods used in the study are non-specific, should be interpreted as possible supplementary tools, and cannot be evaluated independently when making the diagnosis. The disproportion of the number of males and females in the CBS group is a result of the need to exclude patients who did not fulfill all of the examinations planned in the study. Another limitation associated with this investigation is that the increase in familywise error rate across the reported statistical analyses was not controlled. Overall, we consider this research as a pilot study and encourage replication. The aim of the study was to choose tools which could be accessible in everyday practice.

## Conclusions

1. Neuropsychological examinations of patients based on the FAB tool is important and can be helpful in the diagnosis of the subtypes of PSP: PSP-RS and PSP-P.
2. For CBS, SPECT shows greater differences of mean variance values from the neutral level in comparison to PSP-RS and PSP-P. This may be related to a more severe clinical course compared to PSP.
3. Currently, basing on the study group, the multiparametric assessment of patients with PSP-RS, PSP-P, and CBS based on SPECT features and FAB has not achieved greater overall performance than the single-parameter assessment. This implies a need for discerning clinical evaluation of the patient by an experienced clinician during the diagnostic process and use of SPECT and FAB as accessory tools.

## Data Availability Statement

The raw data supporting the conclusions of this article will be made available by the authors, without undue reservation.

## Ethics Statement

The studies involving human participants were reviewed and approved by Ethical Committee of the Medical University of Warsaw. The patients/participants provided their written informed consent to participate in this study.

## Author Contributions

PA study design, data analysis, review of literature, and discussion. BM data analysis, statistical analysis, and discussion. NM data analysis, review of literature, and discussion. KD-W, AD, LK, and AF data analysis and discussion. IC, AS, and MS data analysis. All authors contributed to the article and approved the submitted version.

## Conflict of Interest

The authors declare that the research was conducted in the absence of any commercial or financial relationships that could be construed as a potential conflict of interest.
